# Aberrant pancreas adenocarcinoma in the stomach: A case report and literature review

**DOI:** 10.1097/MD.0000000000032642

**Published:** 2023-01-13

**Authors:** Vidas Petrauskas, Rokas Stulpinas, Ugnius Mickys, Raminta Luksaite-Lukste, Kestutis Strupas, Eligijus Poskus

**Affiliations:** a Clinic of Gastroenterology, Nephrourology and Surgery, Institute of Clinical Medicine, Faculty of Medicine, Vilnius University, Vilnius, Lithuania; b Centre of Abdominal and Oncological surgery, Vilnius University Hospital Santaros Klinikos, Vilnius, Lithuania; c National Centre of Pathology, Affiliate of Vilnius University Hospital Santaros Clinics, Vilnius, Lithuania; d Department of Radiology, Nuclear Medicine and Medical Physics, Institute of Biomedical Sciences, Faculty of Medicine, Vilnius University, Vilnius, Lithuania.

**Keywords:** aberrant pancreas, case report, ductal adenocarcinoma, pyloric obstruction, stomach

## Abstract

Aberrant pancreatic tissue in the gastrointestinal tract is a relatively common finding. However, malignant transformation is extremely rare. Herein, we report a case of ectopic pancreatic ductal adenocarcinoma in the stomach wall.

**Patient concerns:**

A 38 year old male presented with nausea, bloating, abdominal distention and weight loss for 4 months.

**Diagnoses:**

Endoscopy of upper gastrointestinal tract was performed twice with 2 months interval and a stenotic pyloric part was observed with a suspected submucosal lesion. It was sampled both times, however the pathology findings of the mucosal biopsies were unremarkable with no identifiable neoplastic structures. CT scan and MRI was performed and showed a thickened pyloric wall with a submucosal lesion 15 × 15 mm in diameter. Blood levels of tumor markers carcinoembrionic antigen and carbohydrate antigen 19-9 were within a normal range.

**Interventions:**

Pyloric stenosis progressed and the patient underwent a Billroth type I distal gastric resection with D2 lymphadenectomy. Pathologic examination revealed a well differentiated ductal adenocarcinoma arising in the heterotopic pancreatic tissue (Heinrich type III). The resection margins and lymph nodes were free of tumor. The patient received adjuvant chemotherapy with 6 courses of XELOX.

**Outcomes:**

No disease recurrence is reported in 12 months follow-up.

**Lessons:**

Aberrant pancreatic ductal adenocarcinoma in the stomach is a rare finding, however this pathology should be included in the differential diagnosis of gastric submucosal lesion causing pyloric stenosis.

**What Does This Paper Add to the Literature?**

This is the 18th case of well documented aberrant pancreatic ductal adenocarcinoma in stomach. A symptomatic protruding submucosal stomach lesion, even with negative multiple biopsy results, should prompt suspicion of malignancy and early surgical aggressive treatment should be considered.

## 1. Introduction

An aberrant (also called ectopic or heterotopic) pancreas (AP) is defined as a pancreatic tissue with its own vascular, neural and ductal system outside the normal pancreas without any communication with the latter.^[1,2]^ The lesion is usually small (<1 cm in diameter) and asymptomatic.^[3]^ The AP tissue can be found in up to 1.2% of gastrectomy specimens, and even more commonly in autopsy series ranging from 0.55% to 13.7%.^[4]^ The most common locations for AP are the stomach,^[5]^ duodenum^[6]^ and jejunum.^[2]^ The lesion is usually detected endoscopically and presents as a submucosal mass protruding into the lumen.^[1]^

Malignant transformation of AP is extremely rare. Up to date only 17 well documented cases of AP adenocarcinoma in the stomach have been reported in the English language literature.^[7]^ We present the 18th case of this rare disease with literature review of AP adenocarcinoma in the stomach indexed in the PubMed database (up to February 2022). Our case is the first malignant AP adenocarcinoma diagnosed in a Lithuanian patient. Literature review provides insight into this rare pathology and better understanding of clinical findings, treatment and prognosis.

## 2. Case presentation

### 2.1. Chief complaints

A 38 year old male presented to our department with symptoms of intermittent bloating, nausea, abdominal distention in the epigastrium. Symptoms usually presented 1 to 3 hours after a meal. Weight loss of 10 kg over 4 months was also noted.

### 2.2. History of present illness

The patient suffered from previously mentioned symptoms for 2 weeks before he visited local gastroenterologist. During the initial diagnostic workup esophagogastroscopy was performed and a submucosal lesion obstructing the pylorus was observed. It was not possible to pass the endoscope to duodenum. The stenotic pyloric part was sampled suspecting a scirrhous type gastric carcinoma. However, the pathology findings were unremarkable, showing either normal mucosa or a superficial ulceration with surrounding fibrosis, but no identifiable neoplastic structures.

### 2.3. History of past illness

The patient was previously diagnosed with gastroesophageal reflux disease. He denied other chronic diseases, smoking or alcoholism. He was not taking any medication at the time.

### 2.4. Personal and family history

The patient had no significant personal and family history.

### 2.5. Physical examination upon admission

Physical examination revealed abdominal distention, ballottement and mild discomfort during palpation of the epigastrium.

### 2.6. Laboratory examinations

Complete blood count and routine biochemical investigations were unremarkable. Blood tumor marker tests – carcinoembryonic antigen and carbohydrate antigen 19-9 showed normal values.

### 2.7. Imaging examinations

**Figure 1.**
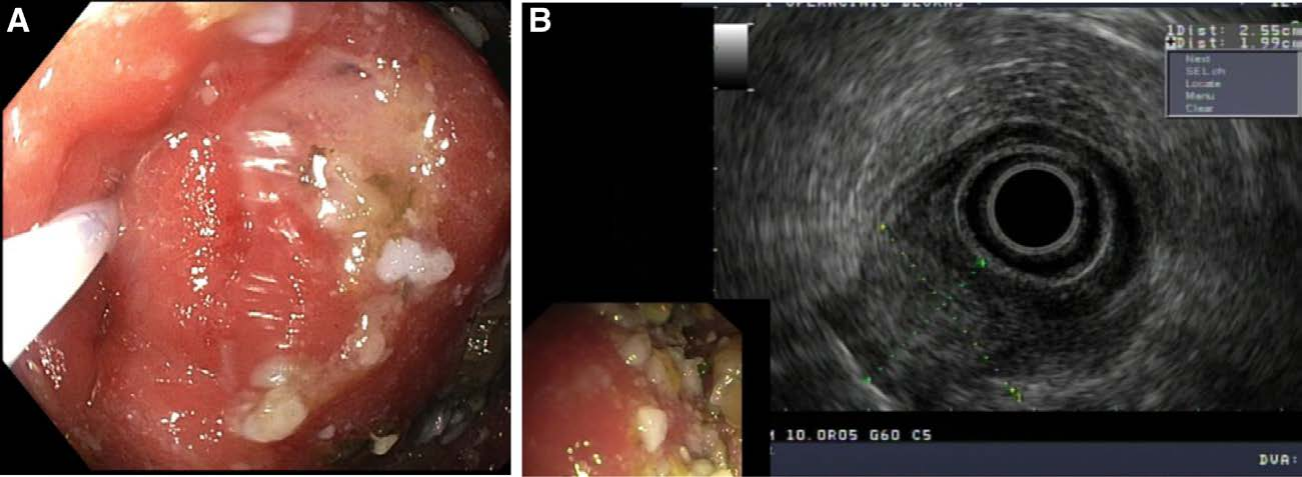
(A) Gastroscopy showing a submucosal lesion with intact mucosa causing pyloric stenosis. Note gastric contents in the lumen and a probe inserted in the stenotic pylorus. (B) Endoscopic ultrasound revealed an isoechoic submucosal lesion in the wall of gastric pylorus.

At our institution esophagogastroscopy was repeated, combined with endoscopic ultrasound, to visualize the lesion. Endoscopy revealed a significant gastric retention (despite previously inserted nasogastric tube) and stenotic pylorus with protruding submucosal lesion in the posterior wall (Fig. 1A). Endosonoscopically a submucosal lesion was identified measuring 2.5 × 1.7 cm in diameter with distorted normal gastric wall anatomy. A 1.5 cm long mucosectomy was performed and 8 deep biopsies from the lesion were obtained (Fig. 1B). Once again no malignant process was identified in the sampled tissue.

**Figure 2.**
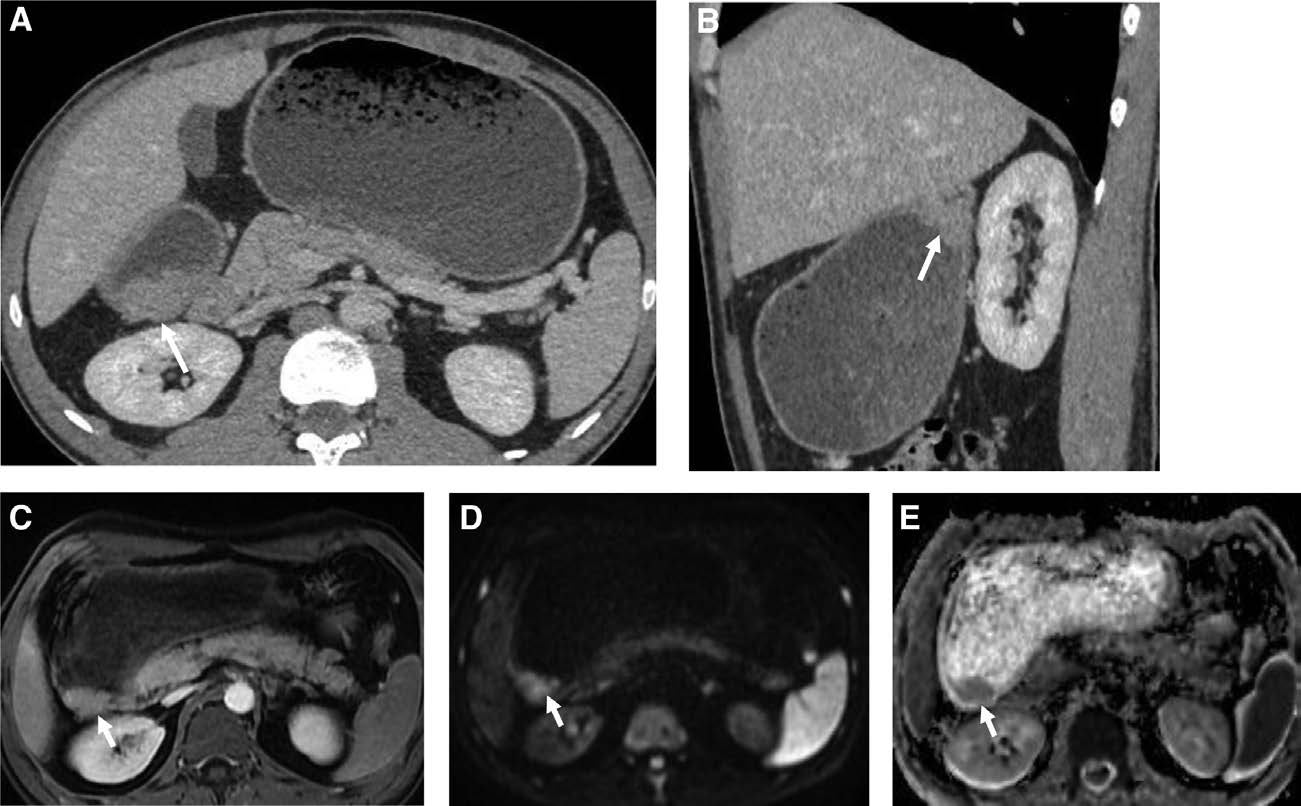
Imaging findings: (A and B) Abdominal CT scan axial and sagittal planes, portovenous phase, show gastrostasis caused by an infiltration of pylorus wall (arrow), (C–E) Abdominal MRI axial plane, there is a contrast enhancing (C) lesion (arrow) with a diffusion restriction (D–E) in the pylorus wall. MRI = magnetic resonance tomography.

Abdominal computed tomography showed marked wall thickening of the pylorus and the lesion was enhanced by contrast agent. Perigastric tissues were not infiltrated and no pathological lymph nodes identified (Fig. 2A and B). Magnetic resonance imaging revealed that there is a contrast enhancing lesion 1.5 × 1.5 cm in diameter with a diffusion restriction in the pylorus wall (Fig. 1C–E). The remainder abdominal scan, including the pancreas, was not significant.

### 2.8. Operation findings

Stenosis progressed and patient underwent operation. During the initial inspection of the abdominal cavity a distended stomach with stenotic pylorus were observed. A fibrous subserosal lesion of the pyloric wall was sampled for frozen section. Ductal type adenocarcinoma (with possible origin of pancreatic tissue) was reported by the pathologist. A decision was made to perform a Billroth type I distal gastric resection with D2 lymphadenectomy (splenic lymph nodes spared).

### 2.9. Pathological findings

**Figure 3.**
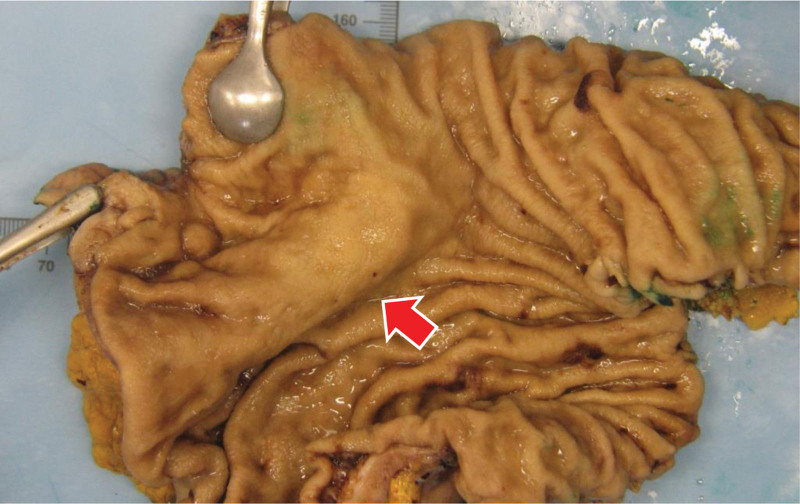
Macroscopic appearance. Flattened, but structurally intact mucosa overlaying the deeper tumor (arrow).

The sample (measuring 18.0 × 8.0 × 4.5 cm after formalin fixation) had intact mucosa with no visually identifiable superficial tumor or defects, but there was an area of flattened folds (Fig. 3) overlaying a 3.0 × 2.1 × 1.3 cm fibrous mass involving the rest of gastric wall.

**Figure 4.**
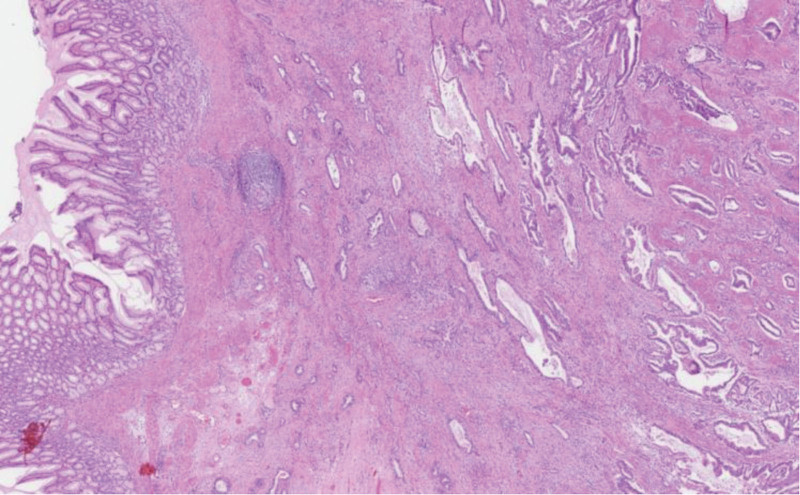
Large duct-like tumor glands in abundant desmoplastic stroma, note the normal pyloric mucosa on the left (hematoxylin-eosin 30×).

**Figure 5.**
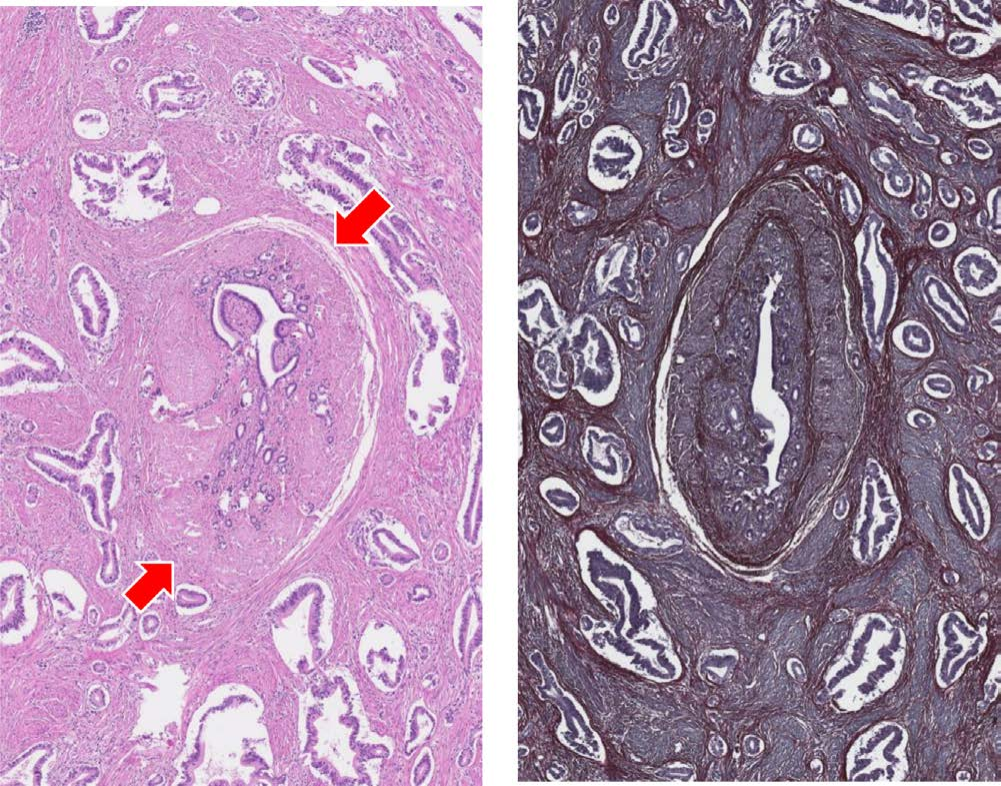
Scattered heterotopic pancreatic ducts could be easily missed among the predominant tumor glands (left: hematoxylin-eosin 70×, right: hematoxylin-orsein 70×).

**Figure 6.**
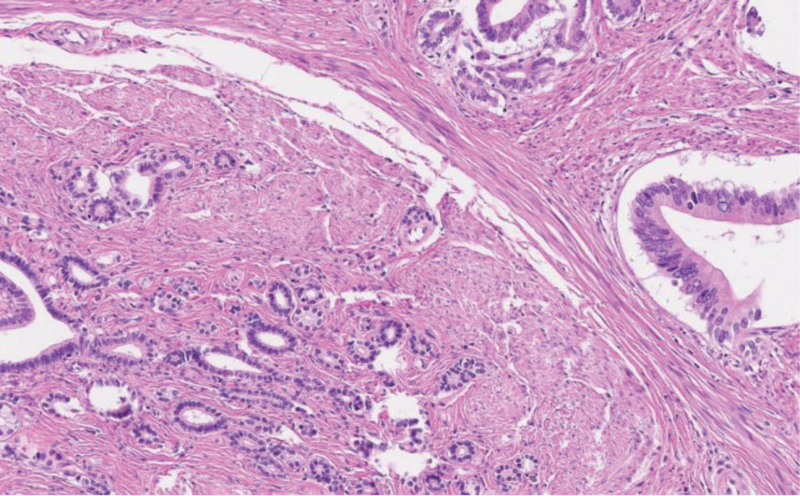
Note the bland epithelium of the heterotopic duct (bottom left) in comparison with malignant glands (top right) (hematoxylin-eosin 200×).

An invasive, vaguely nodular tumor centered in the submucosa and muscular layer with extension into the lesser omentum was seen on microscopic evaluation. Only minimal involvement of the basal mucosa could be identified, explaining the findings of previous biopsies. Tumour was composed of rather large, angulated, duct-like glands, haphazardly infiltrating the abundant desmoplastic stroma (Fig. 4): a pattern not often seen in a primary gastric carcinoma and thus raising suspicion of a secondary spread. The possibility of a pancreatic ductal adenocarcinoma (PDAC) was further heightened by matching (though not specific) immunohistochemical positivity for high molecular weight cytokeratins, CK7, MUC5ac, CDX2, Cadherin 17, and Glypican-3.^[8]^ However, thorough sampling revealed a few nonmalignant pancreatic ducts scattered in between the tumor glands (Figs. 5 and 6). In the setting of normal pancreas (seen on computed tomography [CT] scan and during the operation) this finding leads to a conclusion of a well differentiated ductal adenocarcinoma arising in the heterotopic pancreatic tissue (most likely Heinrich type III – ducts only) in the pyloric wall. Both resection margins and all of the lymph nodes were free of tumor.

## 3. Final diagnosis

The final diagnosis was a well differentiated ductal adenocarcinoma arising in the heterotopic pancreatic tissue (Heinrich type III) in the pyloric wall pT3N0M0, R0.

## 4. Treatment

6 weeks after the surgery the patient received adjuvant chemotherapy with 6 cycles of XELOX (capecitabine, oxaliplatin). No serious adverse events were recorded.

## 5. Outcome and follow-up

At 12 months follow- up no disease recurrence documented (whole body CT, upper gastrointestinal tract endoscopy and biopsies from gastroduodenal anastomosis were negative).

## 6. Discussion

### 6.1. The aberrant pancreas

First ectopic pancreas was described back in 1727 in an ileal diverticulum.^[9]^ AP is not a rare finding reported to be found in 0.5% to 13.7% of autopsies.^[4]^ The most common locations are the stomach (25–60%),^[5]^ duodenum (25–35%)^[6]^ and jejunum (0.5–27%),^[2]^ though different locations have also been reported such as mesocolon,^[10,11]^ Meckel diverticulum,^[12]^ rectum^[13]^ or even biliary system, liver, lung, mediastinum and brain.^[14]^

Three main theories have been proposed to explain the occurrence and possible pathogenesis of AP. The misplacement theory states that, during foregut rotation, several primitive pancreatic elements become separate and are deposited ectopically, forming mature pancreatic tissue. The metaplasia theory proposes that heterotopic pancreas arises from areas of pancreatic metaplasia of the endoderm which migrate to submucosa during embryogenesis. The third theory states that ectopic pancreatic tissue occurring in anatomically remote sites may have different pathogenesis, with the possibility of origin from teratomas.^[1,15,16]^

AP tissue is usually of no clinical importance.^[2]^ If symptoms are present, the site usually influences the clinical manifestation which includes abdominal pain, dyspepsia, bleeding, or obstruction resulting from inflammation or malignancy.^[14,17]^

AP can be found at any age, however it is usually diagnosed in middle-aged group.^[2]^

AP generally appears as a smooth nodule and occasionally as a mass with an irregular surface.^[2]^ It is usually situated in the submucosa and only occasionally expands into the muscularis propria. The mass is broad based, appears to project into the lumen of the stomach or bowel, and is rarely pedunculated.^[2]^ Another possible finding is umbilication as the pancreatic duct opens into bowel lumen.^[2]^ However, this feature is present in less than 45% of cases.^[6]^

Diagnosis is usually challenging due to the lack of specific signs on imaging and multiple non-diagnostic endoscopic biopsies. Some CT findings have been proposed by Liu et al for gastric submucosal mass differentiation (AP vs gastric stromal tumors [GST]) which include location (AP most common localization is body and antrum, whether GST predominantly found in body and fundus), the presence of peritumoral infiltration or fat-line (more common for AP), necrosis (more common for GST) and degree of attenuation (higher Hounsfield units for AP), DEAP [the CT attenuation value of arterial phase minus unenhanced phase] (higher for AP).^[18]^

Only pathological examination of the specimen after surgical resection frequently determines the correct diagnosis.^[1]^

Pathologists further subdivide the AP using a widely accepted Heinrich’s classification.^[19]^ Type I is described as a typical pancreatic tissue with ducts, acini, and islet cells; type II – numerous acini, few ducts, and no islet cells; and type III numerous ducts, few to no acini, and no islet cells.^[19]^ This classification was later modified by Gaspar Fuentes et al,^[20]^ by describing another type of aberrant pancreas (type IV) which consists of endocrine islets without exocrine pancreatic tissue.

### 6.2. Malignancy in aberrant pancreas

The same carcinogenic conditions can affect both the aberrant pancreatic tissue and the normal pancreas. Malignant transformation is a rare event, occurring from 0.7% to 1.8% of all heterotopic pancreata.^[21]^ To our knowledge to date there are only 56 well-documented cases reported of malignancy of AP tissue.^[1,7,22]^ The most common site for malignant transformation is the stomach (35.2%), followed by duodenum (22.2%) and jejunum (14.8%).^[1]^

When AP undergoes a malignant transformation, it is usually symptomatic (84%) with the most common complains being abdominal pain (49%), nausea or vomiting (25%), dyspepsia (15%), weight loss (15%) and gastrointestinal bleeding (8%).^[1,7,22]^

However, upper endoscopy with conventional biopsy often fails to identify a correct cause because the ectopic pancreatic malignancy is usually located deep in the submucosa.^[23]^ Endo et al^[24]^ reported the case of an AP adenocarcinoma diagnosed by endoscopic ultrasound-guided fine-needle aspiration (EUS-FNA). They suggest that EUS-FNA may be useful for the preoperative diagnosis.

The three criteria proposed by Guillou et al^[25]^ are required for a carcinoma to be described as arising from ectopic pancreas:

1. The tumor must be found within or close to the ectopic pancreatic tissue.
2. A direct transition must be observed between pancreatic structures and the carcinoma (malignant transformation of an ectopic pancreas must be differentiated from a metastatic deposit or a neoplastic invasion from a neighboring cancer, especially from the stomach, the biliary tract, and the orthotopic pancreas).
3. The non-neoplastic pancreatic tissue must comprise fully developed acini and ductal structures.

### 6.3. Aberrant pancreatic adenocarcinoma in the stomach

We reviewed literature in English of AP adenocarcinoma in the stomach indexed in the PubMed database up to 2022, February (Table 1). We found 17 well – documented cases. Other case reports were not included in our review, because they were not comprehensive, important information was missing or the pathology report did not fulfill all three criteria proposed by Guillou et al^[25]^ Our case is 18th. Including our case the patients’ age ranges from 31 to 85 years (56.8 on average). There were equal numbers of male and female patients. Most of the patients (89%) were symptomatic with the most frequent presentation of epigastric pain, nausea or vomiting (44%) or gastric distention due to gastric outlet obstruction (33%). Less frequently patients presented with bleeding from gastrointestinal tract (5.6%) or dysphagia (5.6%). In 8 cases no information about tumor markers was reported. Of the remaining 10 cases carcinoembrionic antigen and carbohydrate antigen 19-9 were elevated in 30% of cases^[31,34,36]^ and Ca72-4 elevated only in one case.^[7]^ Majority of the tumors were located in antropyloric region (78%) with submucosal stenotic lesion protruding into the lumen and intact mucosa (61%). Ulceration was described in 5 cases.

**Table 1:** Literature review of aberrant pancreas adenocarcinoma in the stomach.

	**Age (yr), sex**	**Location**	**Macroscopic appearance**	**Symptoms**	**Heinrich type**	**Outcome**	**Size (cm**)	**Tumour markers**
Goldfarb et al,^[26]^ 1962	55, F	Pylorus	Stenotic with ulceration	Epigastralgia, weight loss, vomiting	I	Recurrence (6 yr)	6.0	NR
Tanimura et al,^[27]^ 1979	55, F	Antrum	Submucosal tumor	GI bleeding, gastric discomfort	II	Death (1 mo)	0.5 × 2 × 2	NR
Ura et al,^[28]^ 1997	60, F	Lesser curvature	Extramural mass	Asymptomatic	I	NR	1.8 × 1.3	Normal
Osanai et al,^[29]^ 2001	57, F	Lesser curvature	Protruding lesion with a central ulcer	Epigastric discomfort and periodic nausea	II	Death (13 mo)	12.5 × 9	NR
Halkic et al,^[30]^ 2001	60, M	Esophago-gastric junction	Stenotic, ulcerated	Epigastralgia, dysphagia, weight loss	I	Death (4 mo)	6 × 4.5 × 4	NR
Jeong et al,^[31]^ 2002	64, M	Antrum	Submucosal tumor, stenotic	Dyspepsia, vomiting	I	Uneventful (1 yr)	3 × 3 × 1.5	NR
Song et al,^[4]^ 2004	35, M	Antrum	Submucosal tumor	Asymptomatic	III	Uneventful (5 mo)	2 × 1.7 × 1.2	Normal
Chetty et al,^[15]^ 2004	85, M	Antrum	Ulcer	Dyspepsia, increase in stool frequency	I	Uneventful (1 mo)	1.7 × 1.7 × 0.9	NR
Emerson et al,^[32]^ 2004	52, M	NR	Stenotic	Epigastralgia, vomiting, and bloating	III	Uneventful (9 mo)	4.0 × 2.5 × 1.5	NR
Matsuki et al,^[33]^ 2005	58, F	Prepyloric region	Stenotic	Vomiting	II	Metastasis (1.5 yr)	NR	Normal
Kimura et al,^[34]^ 2008	31, F	Pyloric ring	Submucosal tumor	Epigastralgia	II	NR	5.0 × 2	Elevated
Papaziogas et al,^[35]^ 2008	56, F	Antrum	Ulcerated lesion	Epigastric pain, nausea and vomiting	III	Uneventful (6 mo)	2 × 1.2	Normal
Okamoto et al,^[36]^ 2012	74, F	Middle corpus	Submucosal tumor	Epigastralgia	I	Uneventful (11 yr)	2.0 × 2.0 × 2.0	Elevated
Lemaire et al,^[37]^ 2014	60, M	Lesser curvature	NR	Dyspepsia and epigastric heaviness	NR	Disease-free (4 yr)	7.5 × 4.4	Elevated
Endo et al,^[24]^ 2014	73, M	Antrum	Submucosal tumor	Epigastric pain and abdominal fullness	I	Metastasis (2 yr)	3.2 × 2.4	Normal
Priyathersini et al,^[38]^ 2017	45, M	Antrum	Submucosal mass	Early satiety, vomiting, and constipation	II	Uneventful (12 mo)	5 × 4.3 × 3.5	NR
Xiong et al,^[7]^ 2020	44, F	Antrum	Submucosal tumor, stenotic	Abdominal distension, vomiting	III	Disease-free (9 mo)	3 × 4	Elevated
Present case	38, M	Pyloric ring	Submucosal tumor, stenotic	Nausea, abdominal distention, weight loss	III	Disease-free (12 mo)	3.0 × 2.1 × 1.3	Normal

GI = gastrointestinal; NR = not reported.

Diagnosing malignant transformation of aberrant pancreas in the stomach is a great challenge. Endoscopic biopsy is usually nonspecific, because the lesion is situated deep in the gastric wall. In our case multiple biopsies under ultrasound guidance were obtained, however no neoplastic process was found.

Endoscopic ultrasound (EUS) is suggested for identification of submucosal gastric lesions in order to obtain deep biopsies. EUS was performed for our patient and 5 other cases of the reviewed literature. Malignant lesion evaluated with EUS was suspected in a case reported by Ura et al^[28]^ as an enlarged perigastric lymph node was observed and the mass in the gastric wall changed over time in size and shape. In other cases, including this one, only heterogenous hypoechoic submucosal lesion or thickened gastric wall was observed without any obvious features of malignancy.^[7,24,28,33,37]^

Some authors have suggested that a submucosal gastric lesion of more than 2 cm in diameter should raise suspicion of malignancy.^[35]^ On the other hand, if the lesion is smaller and asymptomatic, no specific treatment is necessary. In our review of the literature, the maximum diameter of the lesion ranged from 1.7 to 12.5 cm (4.1 cm on average) and in one case 2 cm lesion had multiple metastasis to bone and the patient died 1 month after distal gastrectomy because of spread of the disease.^[27]^ Xiong et al^[7]^ suggested that a symptomatic lesion in the gastric submucosa, causing obstruction or weight loss with at least 1 cm in diameter should be treated early and aggressively.

Of all well documented cases of ectopic pancreas adenocarcinoma, the malignancy usually arises from type I heterotopia (46%), followed by type II (33%). In our case the rarest type of heterotopia (III) was observed.

It is not clear what kind of adjuvant chemotherapy regimen should be effective for aberrant pancreatic ductal adenocarcinoma in the stomach.^[7]^ Adjuvant chemotherapy regimen was reported only in three cases of our literature review. These include gemcitabine,^[37]^ S-1 with cisplatin^[24]^ and folinic acid, fluorouracil and oxaliplatin (FOLFOX).^[7]^ Xiong et al reported changing chemotherapy regimen from FOLFOX to gemcitabine because of side effects and increasing tumor marker CA72-4. In our case we selected XELOX with good tolerance and no relapse at 12 months.

It is observed that heterotopic pancreas adenocarcinoma yields a better prognosis (40% were disease free at 1 year of follow up) than orthotopic pancreas carcinoma.^[1]^This is probably related to earlier symptoms and diagnosis. In our literature review majority of the patients were followed up less than 1 year (37.5%) and there were 3 patients (18.7%) disease free at 1 year, and 1 patient (6.2%) did not have recurrence at more than 5 years of follow up. In 6 cases (37.5%) disease recurrence or death was reported. Therefore, it is very hard to draw conclusions on prognosis of aberrant pancreas adenocarcinoma in the stomach, however, the prognosis might be poor.

## 7. Conclusion

Diagnosing aberrant pancreatic adenocarcinoma in the stomach is a great challenge with nonspecific imaging findings and multiple negative biopsies. However, a symptomatic submucosal lesion with progressing abdominal distention should raise suspicion of a malignancy. Early diagnosis with surgical treatment for symptomatic lesions >1 cm in diameter is mandatory to avoid distant spread of the disease.

## Author contributions

VP and EP designed the study. VP wrote the initial draft of the manuscript and literature review. UM and RS wrote the pathologic report. RL evaluated radiologic findings. EP and KS reviewed the manuscript. EP provided critical appraisal of the manuscript. All authors have read and approved the final manuscript.

**Conceptualization:** Vidas Petrauskas, Kestutis Strupas, Eligijus Poskus.

**Data curation:** Rokas Stulpinas, Ugnius Mickys, Raminta Luksaite – Lukste.

**Formal analysis:** Vidas Petrauskas.

**Methodology:** Eligijus Poskus.

**Supervision:** Kestutis Strupas, Eligijus Poskus.

**Validation:** Ugnius Mickys, Raminta Luksaite – Lukste, Eligijus Poskus.

**Visualization:** Rokas Stulpinas, Ugnius Mickys, Raminta Luksaite – Lukste.

**Writing – original draft:** Vidas Petrauskas.

**Writing – review & editing:** Vidas Petrauskas, Eligijus Poskus.

## References

[1] Cazacu IM Luzuriaga Chavez AA Nogueras Gonzalez GM. Malignant transformation of ectopic pancreas. Dig Dis Sci. 2019;64:655–68. 30415408 10.1007/s10620-018-5366-z

[2] Thoeni RF Gedgaudas RK. Ectopic pancreas: usual and unusual features. Gastrointest Radiol. 1980;5:37–42. 6965644 10.1007/BF01888597

[3] Zhang Y Sun X Gold JS. Heterotopic pancreas: a clinicopathological study of 184 cases from a single high-volume medical center in China. Hum Pathol. 2016;55:135–42. 27195908 10.1016/j.humpath.2016.05.004

[4] Song DE Kwon Y Kim KR. Adenocarcinoma arising in gastric heterotopic pancreas: a case report. J Korean Med Sci. 2004;19:145–8. 14966359 10.3346/jkms.2004.19.1.145 PMC2822253

[5] Mulholland KC Wallace WD Epanomeritakis E. Pseudocyst formation in gastric ectopic pancreas. JOP. 2004;5:498–501. 15536290

[6] Mizuno Y Sumi Y Nachi S. Acinar cell carcinoma arising from an ectopic pancreas. Surg Today. 2007;37:704–7. 17643220 10.1007/s00595-006-3384-5

[7] Xiong Y Xie Y Jin DD. Heterotopic pancreas adenocarcinoma in the stomach: a case report and literature review. World J Clin Cases. 2020;8:1979–87. 32518790 10.12998/wjcc.v8.i10.1979 PMC7262711

[8] Yao H Yang Z Liu Z. Glypican-3 and KRT19 are markers associating with metastasis and poor prognosis of pancreatic ductal adenocarcinoma. Cancer Biomark. 2016;17:397–404. 27689616 10.3233/CBM-160655 PMC13020517

[9] Christodoulidis G Zacharoulis D Barbanis S. Heterotopic pancreas in the stomach: a case report and literature review. World J Gastroenterol. 2007;13:6098–100. 18023108 10.3748/wjg.v13.45.6098 PMC4250899

[10] Ishikawa O Ishiguro S Ohhigashi H. Solid and papillary neoplasm arising from an ectopic pancreas in the mesocolon. Am J Gastroenterol. 1990;85:597–601. 2337064

[11] Tornoczky T Kálmán E Jaksó P. Solid and papillary epithelial neoplasm arising in heterotopic pancreatic tissue of the mesocolon. J Clin Pathol. 2001;54:241–6. 11253140 10.1136/jcp.54.3.241 PMC1731386

[12] Koh HC Page B Black C. Ectopic pancreatic-type malignancy presenting in a Meckel’s diverticulum: a case report and review of the literature. World J Surg Oncol. 2009;7:1–5. 19545406 10.1186/1477-7819-7-54 PMC2709896

[13] Goodarzi M Rashid A Maru D. Invasive ductal adenocarcinoma arising from pancreatic heterotopia in rectum: case report and review of literature. Hum Pathol. 2010;41:1809–13. 20869744 10.1016/j.humpath.2010.06.005

[14] Ulrych J Fryba V Skalova H. Premalignant and malignant lesions of the heterotopic pancreas in the esophagus: a case report and review of the literature. J Gastrointestin Liver Dis. 2015;24:235–9. 26114184 10.15403/jgld.2014.1121.242.uly

[15] Chetty R Weinreb I. Gastric neuroendocrine carcinoma arising from heterotopic pancreatic tissue. J Clin Pathol. 2004;57:314–7. 14990608 10.1136/jcp.2003.013557 PMC1770241

[16] Rezvani M Menias C Sandrasegaran K. Heterotopic pancreas: histopathologic features, imaging findings, and complications. Radiographics. 2017;37:484–99. 28287935 10.1148/rg.2017160091

[17] Hickman DM Frey CF Carson JW. Adenocarcinoma arising in gastric heterotopic pancreas. West J Med. 1981;135:57–62. 7257381 PMC1272925

[18] Liu C Yang F Zhang W. CT differentiation of gastric ectopic pancreas from gastric stromal tumor. BMC Gastroenterol. 2021;21:52. 33541287 10.1186/s12876-021-01617-8 PMC7860050

[19] von Heinrich H. Ein Beitrag zur Histologie des sogen akzesso- rischen Pankreas. Virchows Archiv Pathol Arat Bd. 1909;198:392–401.

[20] Gaspar Fuentes A Campos Tarrech JM Fernández Burgui JL. [Pancreatic ectopias]. Rev Esp Enferm Apar Dig. 1973;39:255–68. 4699117

[21] Yamaoka Y Yamaguchi T Kinugasa Y. Adenocarcinoma arising from jejunal ectopic pancreas mimicking peritoneal metastasis from colon cancer: a case report and literature review. Surg Case Rep. 2015;1:114. 26943438 10.1186/s40792-015-0118-1 PMC4648850

[22] Kaneko T Ohara M Okamura K. Adenocarcinoma arising from an ectopic pancreas in the duodenum: a case report. Surg Case Rep. 2019;5:126. 31388774 10.1186/s40792-019-0684-8 PMC6684697

[23] Yasuda K Cho E Nakajima M. Diagnosis of submucosal lesions of the upper gastrointestinal tract by endoscopic ultrasonography. Gastrointest Endosc. 1990;36(2 Suppl):S17–20. 2184080 10.1016/s0016-5107(90)71010-3

[24] Endo S Saito R Ochi D. Effectiveness of an endoscopic biopsy procedure using EUS-FNA and EMR-C for diagnosing adenocarcinoma arising from ectopic pancreas: two case reports and a literature review. Intern Med. 2014;53:1055–62. 24827484 10.2169/internalmedicine.53.1420

[25] Guillou L Nordback P Gerber C. Ductal adenocarcinoma arising in a heterotopic pancreas situated in a hiatal hernia. Arch Pathol Lab Med. 1994;118:568–71. 8192567

[26] Goldfarb WB Bennet D Monafo W. Carcinoma in heterotopic gastric pancreas. Ann Surg. 1963;158:56–8. 14042636 10.1097/00000658-196307000-00011 PMC1408354

[27] Tanimura A Yamamoto H Shibata H. Carcinoma in heterotopic gastric pancreas. Pathol Int. 1979;29:251–7. 10.1111/j.1440-1827.1979.tb03179.x 552797

[28] Ura H Denno R Hirata K. Carcinoma arising from ectopic pancreas in the stomach: Endosonographic detection of malignant change. J Clin Ultrasound. 1998;26:265–8. 9608371 10.1002/(sici)1097-0096(199806)26:5<265::aid-jcu7>3.0.co;2-a

[29] Osanai M Miyokawa N Tamaki T. Adenocarcinoma arising in gastric heterotopic pancreas: clinicopathological and immunohistochemical study with genetic analysis of a case. Pathol Int. 2001;51:549–54. 11472568 10.1046/j.1440-1827.2001.01240.x

[30] Halkic N Nordback P. Soft-tissue images. Malignant degeneration of heterotopic pancreas. Can J Surg. 2001;44:407. 11764870 PMC3692671

[31] Jeong HY Yang HW Seo SW. Adenocarcinoma arising from an ectopic pancreas in the stomach. Endoscopy. 2002;34:1014–7. 12471549 10.1055/s-2002-35836

[32] Emerson L Layfield LJ Rohr LR. Adenocarcinoma arising in association with gastric heterotopic pancreas: a case report and review of the literature. J Surg Oncol. 2004;87:53–7. 15221920 10.1002/jso.20087

[33] Matsuki M Gouda Y Ando T. Adenocarcinoma arising from aberrant pancreas in the stomach. J Gastroenterol. 2005;40:652–6. 16007401 10.1007/s00535-004-1601-9

[34] Kimura J Kaji M Yamamoto S. A case of adenocarcinoma arising from ectopic gastric pancreas with carcinomatous peritonitis. Jpn J Gastroenterol Surg. 2008;41:399–405.

[35] Papaziogas B Koutelidakis I Tsiaousis P. Carcinoma developing in ectopic pancreatic tissue in the stomach: a case report. Cases J. 2008;1:1–6. 18928565 10.1186/1757-1626-1-249 PMC2577107

[36] Okamoto H Kawaoi A Ogawara T. Invasive ductal carcinoma arising from an ectopic pancreas in the gastric wall: a long-term survival case. Case Rep Oncol. 2012;5:69–73. 22611364 10.1159/000335870 PMC3355652

[37] Lemaire J Delaunoit T Molle G. Adenocarcinoma arising in gastric heterotopic pancreas. Case report and review of the literature. Acta Chir Belg. 2014;114:79–81. 24720145

[38] Priyathersini N Sundaram S Senger J. Malignant transformation in gastric pancreatic heterotopia a case report and review of the literature. JOP J Pancreas. 2017;18:73–7.

